# A 6-Year Epidemiological Study of Mandibular Fractures in Traumatic Patients in North of Iran: Review of 463 Patients

**DOI:** 10.29252/wjps.10.1.71

**Published:** 2021-01

**Authors:** Ramyar Farzan, Avishan Farzan, Ava Farzan, Mohammadbagher Karimpour, Mohammad Tolouie

**Affiliations:** 1 Department of Plastic & Reconstructive Surgery, Guilan University of Medical Sciences, Rasht, Iran

**Keywords:** Epidemiology, Mandibular fracture, Trauma

## Abstract

**BACKGROUND:**

Mandibular fracture is considered the second most common facial fracture worldwide. We aimed to evaluate the epidemiology of mandibular fractures in traumatic patients hospitalized at Velayat Teaching Hospital in Rasht, Iran for 6-year.

**METHODS:**

In this retrospective study, all traumatic patients with mandibular fractures admitted to Velayat Teaching Hospital, Rasht, northern Iran for 6-year (2013-18) were enrolled. The data collection tool was a checklist consisting of two parts: demographic information, and injury data. All data were collected through the Hospital Information System (HIS), and analyzed using SPSS software and descriptive and analytical statistics tests.

**RESULTS:**

Overall, 463 hospitalized patients were reviewed. Males had higher frequency than females. The most common accident place was rural roads. The most frequent mechanism of fractures was road accidents. The most common injuries occurred in motorcyclists, followed by car passengers, pedestrians, and cyclists. The highest and lowest frequency of injury occurred in September, and February, respectively. The most common site of fracture was condyle, followed by trunk. In concurrent fractures, the most frequently affected site was maxillary bone, followed by zygomatic bones, orbital, nasal, and frontal bones.

**CONCLUSION:**

The majority of patients with mandibular fractures were young men of working age following motor vehicle accidents. Consequently, the most effective strategy for reducing accidents leading to mandibular fractures is considering all three components of human, environment, and vehicle.

## INTRODUCTION

The advancement of technology and the rising use of motor vehicles and other unexpected accidents have exposed human beings to various traumatic injuries so that we see a significant increase in the extent of maxillofacial injuries today ^1^. The phenomena such as war, traffic accidents, adventurous entertainment, new martial arts, increased quarrel, violence, and occupational accidents have exacerbated various physical injuries including maxillofacial traumas ^2^. 

One of the most important traumas is maxillofacial trauma, of which mandibular fracture is the second most common fracture ^3^^,^^4^, accounting for 36%-59% of all maxillofacial fractures ^5^. These fractures can be one of the common causes of trauma-related disability ^6^.

The prevalence and causes of these types of injuries vary across countries ^7^. In developing countries, traffic accidents are the main cause of mandibular fractures, however, interpersonal violence and physical assault are the most common cause of this kind of fracture in developed countries ^8^^,^^9^. In the United States, the most frequent cause of mandible fractures is motor vehicle accidents and interpersonal violence ^10^. Jaw fractures can occur alone or along with other facial bone fractures ^11^. Mandibular fractures include condylar, coronoid, mandibular angle, mandibular trunk, alveolar, symphysis, and parasymphysis fractures ^2^.

Maxillofacial fractures are among the most common and complex problems in maxillofacial surgery where mandibular bone involvement is more frequent than other facial bones ^12^. The mandible is an important part of the face that has a functional role in speaking, chewing, and swallowing ^13^. This bone is more prone to fracture due to the position relative to skull, so that the mandibular fracture, especially in the condylar region, plays as a defense mechanism and prevents severe trauma to the upper sensitive areas such as the brain and skull ^14^.

Furthermore, mandibular** i**njuries can cause mental distress in patients ^15^. If these fractures are not treated well, they can result in significant functional and aesthetic complications, including facial asymmetry, dental mismatch, temporomandibular joint disorders, and osteomyelitis ^16^. The incidence and etiology of mandibular trauma can be affected by social, cultural, environmental, and political factors, geographical variation, population density, economic status, regulatory rules including speed limits, mandatory use of seat belts and helmets ^17^. Despite the high mortality rate and disability caused by maxillofacial fractures, few studies have been conducted in Iran. Iran is a large country with diverse ethical, cultural and environmental characteristics; therefore epidemiological studies in specific areas cannot be generalized to all geographical parts of the country ^18^. Since understanding the epidemiology of mandibular fractures is critical for effective prevention, it is necessary to examine the variables such as injury mechanism, injury patterns, and other effective factors ^16^to provide health planners with a clear comprehension of mandibular fracture patterns ^17^.

We aimed to evaluate the 6- year epidemiological study of mandibular fractures in trauma patients admitted to Velayat teaching hospital, as a maxillofacial trauma referral center, in the northern city of Rasht. 

## MATERIALS AND METHODS

This was a retrospective case-series study. All traumatic patients with mandibular fractures (ICD10 code admitted to Velayat Referral Center of Maxillofacial Trauma in Rasht, northern Iran were enrolled in the study for 6 years (2013-18) through census method. The samples’ CT scans were examined by an oral and maxillofacial radiologist. The data collection tool was a checklist consisting of two parts: demographic information (name, age, and gender); and injury data (cause of trauma, place and time of accident, site of fracture, concurrent fractures in other facial parts, injury to other organs, the time interval between hospitalization and surgery, and comorbid early complications). Patients discharged against medical advice, patients with mild fractures of mandible, patients with no definite diagnosis using routine radiographic imaging, and patients with incomplete information in the medical records were excluded from the study. All data were collected through Health Information System (HIS), and analyzed using SPSS software version 21 and descriptive statistics tests.

The study was proved by local Ethics Committee with Ethical Code: IR.GUMS.REC.1396.389.

## RESULTS

The medical records of 463 patients were reviewed. Men had higher frequency than women (n=408; 88.1%). The mean age of the subjects was 29.79±14.21 years. The youngest and oldest subject was 2 yr and 81 yr old, respectively. The most common cause of mandibular fractures was road crashes (n=386, 83.4%). The most frequent places of accident were rural roads (61.3%). The most frequent cause of fractures was road-related accidents (83.4%). These accidents caused injury in 89.1% of men and 10.9% of women. Following road-related accidents, falls (7.1%) were the second most common mechanism of fractures. Motorcyclists were affected most frequently by road accidents (n=241; 62.4%), followed by car passengers (27.7%), pedestrians (8.3%), and cyclists (1.6%) (Table 1). Men had the highest percentage in all mechanisms of accidents. No statistically significant relationship was seen between gender and mechanism of accident (*P*=0.353) (Table 2). The highest and lowest frequency of injury happened in Sep and Feb, respectively (13.6% vs. 2.6%) (Table 3). 

**Table 1:** Studying Gender, Accident Place, and Use Variable (Number and Frequency Percentage)

**Variable**		**Number**	**%**	**Variable**	**Number**	**%**
Gender	Woman	55	11.9			
	Man	408	88.1			
Road Crash Place	Rural Roads	284	61.3			
	Interurban Roads	102	22.03			
Accident Mechanism	Road Crashes	386	83.4	Car	107	27.7
				Motorcycle	241	62.4
				Pedestrian	32	8.3
				Bicycle	6	1.6
	Workplace Accidents	6	1.3			
	Quarrel	23	5			
	Falls	33	7.1			
	Sports Accidents	15	3.2			

**Table 2:** The Frequency Distribution of Gender in Accident Mechanisms

**Gende**r	**Man**		**Woman**	
Accident Mechanism	Number	%	Number	%
Road Crashes	344	89.1	42	10.9
Workplace Accidents	5	83.3	1	16.7
Quarrel	21	91.3	2	8.7
Falls	26	78.8	7	21.2
Sports Accidents	12	80	3	20

**Table 3:** The Frequency Distribution of the Accident Time (Months)

Variable		Number	%
Accident Time	April	51	11
	May	48	10.4
	June	55	11.9
	July	51	11
	August	61	13.2
	September	62	13.4
	October	35	7.6
	November	32	6.9
	December	27	5.8
	January	15	3.2
	February	12	2.6
	March	14	3

Overall, 688 anatomical fracture sites were observed in 463 patients. The most common fracture site was condyle (n=164; 35.4%), followed by trunk (n=162; 35%) (Table 4). The most frequent cause of condylar fractures was reported road accidents (n=130; 73.3%), followed by falls (n=17; 10.4%). The most common cause of trunk fractures was road accidents (n=138; 85.2%), followed by falls (n=10; 6.2%).

**Table 4:** Frequent Distribution of Fracture Site in Traumatic Patients with Mandibular Fractures by Gender and Place of Accident

Fracture Site	Frequency		Gender				Accident Place			
			Man		Woman		Urban		Rural	
	Number	%	Number	%	Number	%	Number	%	Number	%
Symphysis	51	11	44	86.3	7	13.7	16	31.4	35	68.6
Parasymphysis	117	25.3	104	88.9	13	11.1	41	35	76	65
Condyle	164	35.4	145	88.4	19	11.7	54	32.9	110	67.1
Coronoid	2	0.4	2	100	0	0	0	0	2	100
Ramus	8	1.7	7	84.5	1	12.5	0	0	8	100
Trunk	162	35	143	88.3	19	11.7	44	27.2	118	72.8
Alveole	50	10.8	43	86	7	14	11	22	39	78
Angle	134	28.9	120	89.6	14	10.4	42	31.3	92	68.7

In addition to mandibular fractures, 127 patients (27.4%) suffered from fractures in other facial bones (297 fractures). The most frequent fracture occurred in maxillary bone (n=98; 32.9%), followed by zygomatic (n=79; 26.6%), orbital (n=78; 26.4%), nasal (n=34; 11.4%), and frontal bone (n=8; 2.7%) (Table 5).

**Table 5:** Frequency Distribution of Facial Bones Concurrent Fractures in Traumatic Patients with Mandibular Bone Fracture

**Facial Bones**	**Number**	**%**
Maxillary	98	32.9
Zygomatic	79	26.6
Orbital	78	26.4
Nasal	34	11.4
Frontal	8	2.7
Total	297	100

In addition, concurrent injuries to other organs were seen in 53 cases (11.4%) (77 concurrent injuries in 53 cases). These injuries included head injury (n=46; 59.7%), upper and lower extremities (n=12; 15.6%), vertebral column and thorax (n=7; 9.1%), and abdomen (n=5; 6.5%) (Table 6).

**Table 6:** Study of the Frequency Distribution of Comorbid Injuries in Traumatic Patients with Mandibular Fracture

**Comorbid Injury Site**	**Number**	**%**
Head	46	59.7
Extremities	12	15.6
Vertebral Column	7	9.1
Thorax	7	9.1
Abdomen	5	6.5
Total	77	100

The mean time from admission to surgery in patients with mandibular fractures was 3.52±5.01 days. The maximum and minimum time was 54 d and less than one day, respectively). 

## DISCUSSION

One of the major problems in traumatic patients is maxillofacial fractures ^19^. Failure to diagnose and treat facial fractures promptly can lead to developmental abnormalities, facial deformities, and teeth loss ^20^. According to the preliminary results of the present study, the frequency of injury was higher in men than that in women, and the mean age of the participants was 29.79±14.21 yr old. This is consistent with other studies’ results ^21^^-^^23^. Since the second and third decades of age are the most active period of human life ^24^^, ^^25^, and men have more work activities at the communities, and because of the major etiology of fractures and moods of men for risk-taking, these results are expected^26^^-^^28^. Of course, the cultural, social and economic conditions of any society can affect that. It is expected to increase the likelihood of these injuries in women when creating working opportunities for women in developing societies nowadays ^1^.

The current study on the causes of mandibular trauma found that the most common causes of injury were road accidents, followed by falls, quarrels, workplace accidents, and sports accidents. There are various studies to support these results ^29^^-^^31^. High numbers of road traffic accidents are caused by the factors such as lack of highway capacity and road black spots, unsafe roads with inadequate engineering structures, vehicles lacking safety features, unauthorized speeding, and traffic rules violations ^27^^,^^29^. Because of geographical and touristic position and heavy traffic of motor vehicles, Guilan Province suffers from high road accidents and therefore related traumas.

There are, however, other studies that contradict our results that falls and sporting accidents have high frequency ^30^^-^^33^. This can be due to the various social, cultural, political, and economic structures in different societies.

In this study, motorcyclists were the most frequent road users affected by jaw trauma. In other similar studies, motorcyclists had the most common numbers ^21^^,^^22^. According to WHO statistics, motorcyclists are one of the most vulnerable road users ^29^. The unsafe nature of motorcycle and its rising use by young people is associated with an increased incidence of traffic accidents ^34^. In general, the increase in the number of motorcycles and related injuries throughout the country are associated with the factors such as the decline in motorcycle prices, the disproportionate growth in the quantity and quality of public transportation vehicles, rising citizens' expectations and earnings, the use of motorcycles as a means to reach workplace and living place, the limitations and disadvantages of motorcycle and loss of effective measures to improve the safety of motorcycling, and the use of safety equipment including helmets, clothing, shoes, gloves, glasses and knee strap. Furthermore, geometric design and road maintenance are usually tailored to the needs of vehicles such as cars and trucks, while motorcycle requirements are often ignored. One reason may be the loss of experience or awareness of the engineers and departments’ authorities in a comprehensive view of all road vehicles ^35^.

The current study showed that most injuries occur in the first half of the year mostly in summer, especially in September. Most cases of maxillary trauma occur in summer in Turkey ^8^. Due to the spring and summer holidays and the high frequency of travel trips during these times, road accidents and traumas have a high prevalence.

The accident mostly occurred on rural roads rather than urban ones, as motorcycle is an important vehicle in transportation in narrow and non-standard rural roads, and plays a significant role in agricultural affairs in Guilan Province, and consequently the results were expected (given that most traumas occurred in the first half of the year when agricultural work is mostly conducted).

The most common anatomical site of fracture in our study was the condyle, which was mostly seen in traffic accidents in men, followed by trunk fracture. According to the results of similar studies, condyle was the most frequent site of injury in traumas ^8^^, ^^22^^, ^^23^. Other studies, however, suggest that condylar fractures is not always the most common ^4^^, ^^8^^, ^^14^^, ^^17^^, ^^22^^, ^^28^^-^^35^.

The present study showed that 27.4% of cases suffer from mandibular bone fractures in addition to other facial bones fractures including maxillary bones, followed by zygomatic, orbital, nasal, and frontal bones. Moreover, 11.4% of the traumatic patients with mandibular fracture suffered from concurrent injuries to other organs. The most significant injury was head trauma. Mandibular fractures and injuries such as skull fractures, or subarachnoid hemorrhage, and subdural hemorrhage happened concurrently ^22^. Zygomatic fractures were most prevalent along with mandibular fractures, and 36% of patients had concomitant injuries to other organs especially skull ^8^. The most frequent site of fractures in maxillofacial trauma was mandibular bone (27.3%), followed by zygomatic (20.9%), and nasal bone (18.8%) ^7^. Various studies have documented different frequencies for facial bone fractures and concurrent injuries to other organs, but head trauma had the most frequency in all of them. 

Most traumas are seen in young male motorcyclists, unauthorized speeds, showing-off driving, and not using appropriate safety equipment such as helmets play an important role in head trauma ^29^. Considering the importance of early complications including hemorrhage, hematoma, airway injury, and respiratory dysfunction, and the necessity of timely diagnosis and emergency treatment for patients with mandibular trauma, studies revealed that none of the patients had any early complications, fortunately.

## CONCLUSION

The majority of people with mandibular fractures were young men of working age following motor vehicle accidents, and most injuries were due to driving accidents on rural roads. Therefore, motorcyclists must be preferred in safety programs for rural roads. Thereby, adopting stricter rules and policies for motorcyclists compared to other drivers can be effective in reducing accidents in these areas. Moreover, the most efficient strategy for reducing all human driving accidents is considering all three components of human, environment, and vehicle. Therefore, the importance of adhering to safety principles through periodic, formulated, and appropriate education to the age groups, and through competent authorities, is emphasized.
